# Does mechanical loading restore ligament biomechanics after injury? A systematic review of studies using animal models

**DOI:** 10.1186/s12891-023-06653-x

**Published:** 2023-06-22

**Authors:** Chris Bleakley, Fredh Netterström-Wedin

**Affiliations:** 1 School of Health Sciences, Faculty of Life and Health Sciences, Ulster University, Jordanstown campus, Newtownabbey, UK; 2 Division of Public Health Science, School of Health Sciences, Mid Sweden University, Sundsvall, Sweden; 3 School of Public Health and Community Medicine, University of Gothenburg, Gothenburg, Sweden

**Keywords:** Ligament, Injury, Mechanical loading, Exercise, material properties, Stiffness

## Abstract

**Background:**

Mechanical loading is purported to restore ligament biomechanics post-injury. But this is difficult to corroborate in clinical research when key ligament tissue properties (e.g. strength, stiffness), cannot be accurately measured. We reviewed experimental animal models, to evaluate if post-injury loading restores tissue biomechanics more favourably than immobilisation or unloading. Our second objective was to explore if outcomes are moderated by loading parameters (e.g. nature, magnitude, duration, frequency of loading).

**Methods:**

Electronic and supplemental searches were performed in April 2021 and updated in May 2023. We included controlled trials using injured animal ligament models, where at least one group was subjected to a mechanical loading intervention postinjury. There were no restrictions on the dose, time of initiation, intensity, or nature of the load. Animals with concomitant fractures or tendon injuries were excluded. Prespecified primary and secondary outcomes were force/stress at ligament failure, stiffness, laxity/deformation. The Systematic Review Center for Laboratory animal Experimentation tool was used to assess the risk of bias.

**Results:**

There were seven eligible studies; all had a high risk of bias. All studies used surgically induced injury to the medial collateral ligament of the rat or rabbit knee. Three studies recorded large effects in favour of ad libitum loading postinjury (vs. unloading), for force at failure and stiffness at 12-week follow up. However, loaded ligaments had greater laxity at initial recruitment (vs. unloaded) at 6 and 12 weeks postinjury. There were trends from two studies that adding structured exercise intervention (short bouts of daily swimming) to ad libitum activity further enhances ligament behaviour under high loads (force at failure, stiffness). Only one study compared different loading parameters (e.g. type, frequency); reporting that an increase in loading duration (from 5 to 15 min/day) had minimal effect on biomechanical outcomes.

**Conclusion:**

There is preliminary evidence that post-injury loading results in stronger, stiffer ligament tissue, but has a negative effect on low load extensibility. Findings are preliminary due to high risk of bias in animal models, and the optimal loading dose for healing ligaments remains unclear.

**Supplementary Information:**

The online version contains supplementary material available at 10.1186/s12891-023-06653-x.

## Background

Lower-limb ligament injuries commonly occur in sports. There is a high incidence of collateral ligament tears to the knee or ankle in contact sports such as ice hockey, [1] American football, [2] soccer, [3, 4] or rugby union [5, 6]. Prospective data estimates 0.11 knee medial collateral ligament (MCL) injuries per 1000 athlete-exposures (AEs) in intercollegiate athletics, [7] and 1.18 ankle sprains per 1000 AE in field sports [8]. Recurrence rates are high with up to 40% of patients developing chronic ankle instability (CAI), [9] and 11% [3] to 16% [10] suffering recurrent knee MCL injury.

Apart from ACL tears, most lower limb ligament injuries are managed conservatively through progressive mobilisation and therapeutic exercise. Prospective interventional research is lacking for knee MCL, but meta-analyses show exercise-based rehabilitation improves function and reduces reinjury after ankle sprain compared to immobilisation [11–13]. These positive effects are largely attributed to the resolution of clinical impairments, with therapeutic exercise restoring joint range of motion, strength, postural control and global movement patterns [14]. An under-discussed mechanism of effect is that post-injury exercise directly enhances ligament healing. This is underpinned by mechanotransduction, where biophysical forces imparted on healing tissues (due to exercise), are converted to cellular and molecular responses, [15] ultimately restoring tissue mass and mechanics. The ability to use exercise to increase ligament strength post-injury has significant implications for rehabilitation programs. However, these effects are most likely dose-dependent, with ‘optimal loading’ of injured ligament tissue, defined as the load applied to structures that maximises physiological adaptation [16].

Identifying the mechanism of action for physiotherapeutic interventions can help to determine the optimal dose, and potentially which patients are most likely to respond to treatment. Available clinical data cannot delineate the effects of progressive loading on ligament healing. No clinical studies have stringently manipulated key loading variables (e.g. nature, magnitude, duration, frequency of loading) [16]; and outcomes measures are primarily limited to binary or ordinal assessment of mechanical joint stability (e.g. stable vs. unstable). In animal models, the research can manipulate loading parameters post-injury, and directly quantify tissue mechanics such as ligament stress, strain, ultimate tensile strength, and stiffness. By systematically reviewing the animal literature we determine the extent to which mechanical loading (and its constituent parameters) can restore ligament biomechanics post-injury, and better understand the mechanisms of rehabilitation after soft tissue injury.

In this review, we examined the biomechanical effects of loading in injured animal ligament models. Our objectives were to determine if mechanical loading interventions restore tissue biomechanics (e.g. tensile strength; stiffness) more favourably than immobilisation or unloading and to explore if outcomes are moderated by loading parameters (e.g. nature, magnitude, duration, frequency of loading).

## Methods

### Protocol and registration

This systematic review was conducted and reported according to Preferred Reporting Items for Systematic Reviews and Meta-Analyses (PRISMA 2020) [17] and was prospectively registered to PROSPERO on 30 November 2020 (registration number: CRD42020210679).

### Eligibility criteria

We included published controlled or randomised controlled trials using an injured animal ligament model. Animals with concomitant fractures or tendon injuries were excluded. Eligibility criteria were outlined as follows:

- *Intervention*: At least one group must have been subjected to a mechanical loading intervention post-injury, with no restrictions on the dose, time of initiation, intensity, or nature of the load. Studies with several different loading arms were also considered, to discern whether differing loading protocols could influence outcomes. Interventions that used surgical repair were excluded.
- *Comparator*: Comparisons could have been made to strict immobilisation or hindlimb suspension. The lowest load group was considered the comparator if several different loading arms were studied. Comparisons that used surgical repair were excluded.
- *Outcomes*: Outcomes could have involved any type of biomechanical measures related to soft tissue healing, but both primary (failure force; failure stress) and secondary (stiffness; laxity or deformation) outcomes were explicitly prespecified.

We applied no language restrictions. The inclusion and exclusion criteria are summarised in Table 1.

**Table 1:** PICOTS criteria for inclusion and exclusion of studies

Parameter	Inclusion criteria	Exclusion criteria
**P**roblem	▪ Animal model ▪ Surgically induced ligament injury at a weight-bearing joint	Fractures, tendon injuries
**I**ntervention	Any physical activity or exercise-based treatment protocol	Surgical repair
**C**omparator	Immobilisation, hindlimb suspension	Surgical repair
**O**utcome measure	▪ Primary: ligament force and stress at failure ▪ Secondary: laxity and deformation at failure ▪ Tertiary: any other outcome related to tissue biomechanics	
**T**ype of study	Controlled trial	Case studies; case series
**S**etting	Laboratory based	

### Search strategy

Electronic database searches were undertaken through OvidSP and ProQuest, using the following databases: MEDLINE and EMBASE (searched from inception throughout on April 28, 2021) and the Agricultural & Environmental Science Collection (searched from inception throughout on April 23, 2021). Updated searching was performed on May 29, 2023. The search strategy used for MEDLINE is outlined in Table 2.

**Table 2:** MEDLINE search strategy

1	(protection or rest or functional or movement or exercise or loading or unloading or weight-bearing or weight-bear or cast or splint or immobilisation or immobilization).mp.
2	exp Ligaments/
3	exp “Sprains and Strains”/
4	2 or 3
5	1 and 4
6	Limit 5 to animals

We also performed supplementary searching, which included:

- Searching PubMed using the “see all similar articles” search function (May 12, 2021) for all studies meeting the inclusion criteria.
- (forward) citation tracking using PubMed citation index function (May 29, 2023).
- Hand-searching the reference lists of all articles meeting the inclusion criteria of our systematic review.

Full details regarding all searches are available in Supplementary File 1.

Two reviewers (CMB, FN-W) independently screened the titles and abstracts of all records produced from the electronic and supplementary searches. Full-text versions were retrieved for any potentially relevant articles. In case of disagreement, both reviewers discussed until a consensus was reached. Since we encountered no ambiguity regarding potential inclusion eligibility, there was no need to contact any corresponding authors.

### Study risk of bias assessment

Both authors performed an independent assessment of included studies using the SYstematic Review Center for Laboratory animal Experimentation (SYRCLE) risk of bias tool [18]. SYRCLE is a 10-item scale covering the following domains: (1) sequence generation; 2) baseline characteristics; 3) allocation concealment; 4) random housing; blinding of 5) caregivers, and 6) outcome assessors; 7) random outcome assessment; 8) incomplete outcome data; 9) selective outcome reporting, and; 10) other sources of bias. We determined the overall risk of bias for each study based on its highest level of bias in any single domain. Both authors reached complete agreement following a consensus meeting, negating the need for a third assessor.

### Data items

The primary author (CMB) extracted the following data items from the eligible studies into a pre-defined data form:

- Study characteristics (study design, number and size of eligible groups, nature of the comparison (e.g., cage activity vs. immobilisation; low dose exercise vs. high dose exercise).
- Animal model (type of animal and their age, sex and weight, injury mechanism (size of ligament injury), joint and ligament(s) affected).
- Treatment interventions (method, dose, duration).
- Biomechanical outcomes: including the results of testing, the unit of measurement, and follow-up times (weeks post-injury).
- Biomechanical testing methods (preconditioning before testing, joint position during loading, and the type of load applied during outcome assessment).

Sufficient information was available to negate the need to contact any original study authors. Data (mean, SD) were extracted from graphs using WebPlotDigitizer (version 4.4) when required. The second author (FN-W) reviewed and confirmed the extracted information against the original research reports.

### Data synthesis

The lead author (CMB) extracted relevant outcome data (mean, SD, number of subjects) into RevMan 5.4. For each study, differences in means between groups and the associated 95% confidence intervals were calculated for continuous outcomes. Since the continuous outcomes were measured on different scales in different studies, we used standardised mean differences (SMD). The specific type of SMD calculated in Revman 5.4 is Hedges *g*: SMDs of 0.2, 0.5, and 0.8 are considered small, moderate, and large magnitudes of effect, respectively [19]. For studies that reported the standard error with the group means instead of the standard deviation; we converted the reported standard error into a standard deviation by multiplying the standard error with the square root of the corresponding group size. We calculated risk ratios (RRs) for dichotomous variables, using a 0.5 zero-cell correction in the case of no events.

In studies that had both concomitant and isolated ligament injury models, we only extracted data for the latter, as widespread injuries are less likely to be treated conservatively in clinical practice [20]. We had planned to preferentially extract data based on changes from baseline (mean change scores). However, this was not reported in any study, and follow-up scores were used instead. We prespecified that if studies included multiple observations of the same outcome, we would extract outcome measures at four-week intervals post-injury, with no limit on follow-up duration (none of the included studies coincided precisely with four-week intervals, necessitating less stringent criteria). Although some studies induced bilateral scarring and performed biomechanical assessments of both knee joints, we treated the sample size as the number of subjects rather than the number of knee joints in our analyses. Our primary and secondary outcomes are presented in forest plots. Studies with similar follow-up periods were grouped.

The second author (FN-W) reviewed the primary author’s (CMB) extracted outcome data, either comparing it to the original research reports or – if data were only presented in graphs – replicating the graphical data extraction process. Any discrepancies between the two authors were settled through consensus.

## Results

### Study selection

**Fig. 1:**
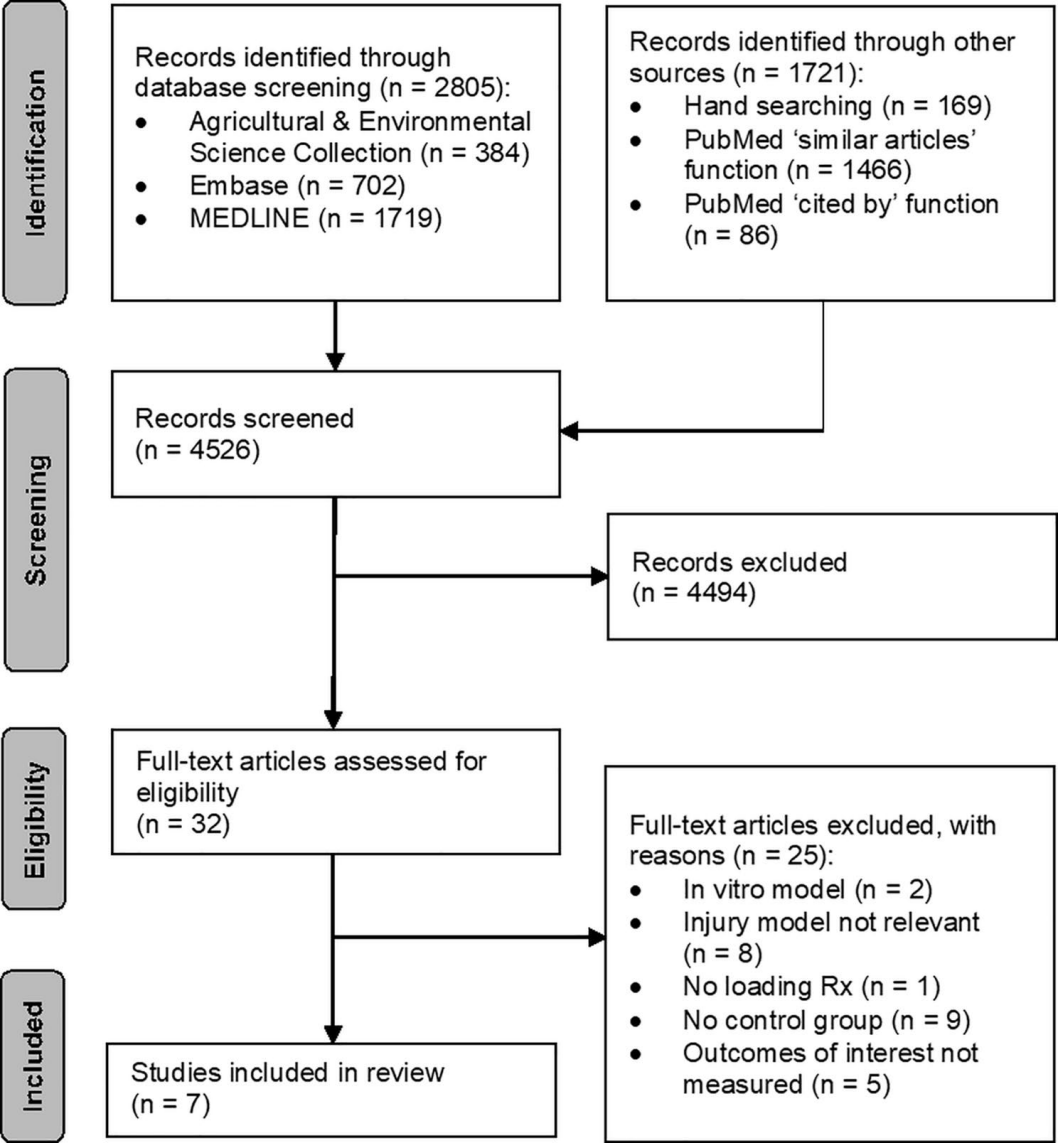
Flow chart of search strategy

The study selection process is outlined in Fig. 1. We identified and examined 4526 records. Of the 32 full-text articles assessed for eligibility, 25 were excluded due to: no control group, [21–28] [29] injury model not relevant, [30–37] outcomes of interest not measured, [38–42] in vitro model, [43, 44] and no relevant intervention [45]. In total, seven studies [46–52] were included in this systematic review.

### Study characteristics and results

Study characteristics are presented in Table 3. There were seven eligible studies: one randomised controlled trial [46] and six non-randomised controlled trials [47–52]. All used rat [46, 50, 51] or rabbit models, [47–49, 52] with surgically induced injury to the MCL at or just below the knee joint line. Animals were either all female [47–49, 52] or all male [46, 50, 51]. Only two studies [47, 48] specified the animals’ age (1 year) at the time of testing. Three studies specified that the length of the ligament transection was 4 mm [47–49]. Two studies also transected the ipsilateral ACL [47] or the ACL plus medial capsule [51]. Biomechanical testing was undertaken at various time points post-injury, with group sizes ranging from 2 to 10 specimens, depending on the study and sacrifice interval.

In all studies, biomechanical testing involved tensile loading of the isolated MCL until failure, with the knee joint positioned in 45 to 90 degrees of flexion. The load was either applied cyclically [48, 49, 52] or continuously, [46, 47, 50, 51] but there was variation in the loading strain rates. (Table 3) Four studies [46–49] specified the use of ligament preconditioning with low loads, prior to final outcome testing.

Five studies [46, 47, 49–51] recorded force at failure; these data were expressed in absolute units (N or N/kg) in all but one study, [51] when force at failure was reported as a percentage of an uninjured control ligament. Two studies [46, 47] also provided details on ligament stress at failure. Secondary outcomes included ligament stiffness [46, 47, 49–51] and laxity [47, 48, 50–52]. In all studies, stiffness represents the slope of the load-deformation (stress/strain) curve recorded during tensile loading of the healing ligament. In most studies, laxity outcomes reflect ligament tissue response at low tensile loads, quantified by the amount of ligament displacement that occurs before initial restraint. Only Thornton et al. [49] assessed laxity at higher tensile loads, quantifying ligament displacement before complete failure. Tertiary outcomes described were strain [46] and energy [49] at failure, average stress during cyclic testing, [50] and number of failure events during creep testing [48].

Treatment interventions were classified as either unloading, lower load, or higher load. Unloading involved either hind limb suspension [46] or pinning of the injured leg in maximal knee flexion [47–49]. All studies included a lower load intervention based on free cage activity. Interventions classified as higher load were based on free cage activity plus an additional loading mechanism; this was either accomplished through daily swimming, [50, 51] or an intra-articular pin that exerted a tensile load on the healing ligament [52]. In each study, all respective interventions were continued up to the time of biomechanical testing.

**Table 3:** Characteristics of the Included Studies

		Intervention		Outcomes	
Lead Author (Year)	Animal and Injury/Surgery Characteristics	Protection	Low Load, High load	Testing details: Tibiofemoral position and load type (magnitude; rate)	Outcome measures (Follow-up)
Bray (1992) [47]	▪ 46 female rabbits (37 analyzed) ▪ Age, 12 months ▪ BW (total sample): 4.7 ± 0.66 kg ▪ Water ad libitum and similar diets ▪ Knee MCL injury ▪ Surgical, 4-mm midsubstance gap ▪ Ipsilateral ACL fully transected	Immobilization (leg pinned at ~ 150°-160° flexion)	▪ Low: Ad libitum cage activity ▪ High: NA	▪ ~70° flexion ▪ Tensile to failure (20 mm/min)	1. Force at failure (N) 2. Stress at failure (MPa) ^b^ 3. Stiffness (N/mm) ^c^ 4. Laxity (mm)^a^ (3, 6 and14 wk)
Burroughs (1990) [50]	▪ 50 male rats (30 analyzed) ▪ BW for extracted groups: 625 ± 68 g (control); 624 ± 65 g (5-min swim); 626 ± 57 g (15-min swim) ▪ Knee MCL injury ▪ Surgical, bilateral transection just below joint line	NA	▪ Low: Ad libitum cage activity ▪ High: Ad libitum cage activity and swimming^e^ (5 or 15 min per day)	▪ 45° flexion ▪ Tensile to failure (0.25 mm/s)	1. Force at failure (N/kg) 2. Stiffness (N/mm) 3. Laxity (mm)^a^ (12 d)
Gomez (1991) [52]	▪ 24 female rabbits (24 analyzed) ▪ BW (total sample): 3.3 ± 0.11 kg ▪ Knee MCL injury ▪ Surgical, transection	NA	▪ Low: Ad libitum cage activity ▪ High: Ad libitum cage activity (4 wk) plus tensile stress (intra-articular pin insert remaining in situ up to the time of biomechanical testing)	▪ 90° flexion ▪ Cyclic (0–4% strain)	1. Average stress during cyclic loading 2. Stiffness (between 3 and 4% strain) (MPa) 3. Laxity during varus to valgus^ab^ (6 and 12 wk)
Lechner (1991) [51]	▪ 40 male rats (25 analyzed) ▪ BW (total sample): range, 400 to 600 g ▪ Knee MCL, ACL, and medial capsule injury ▪ Surgical, transection	NA	▪ Low: Ad libitum cage activity ▪ High: Ad libitum cage activity plus swimming (per day: 10 min swim, 5 min rest, 10 min swim)	▪ 45° flexion ▪ Tensile to failure (0.25 mm/s)	1. Force at failure ^b^ (% of control ligament) 2. Stiffness ^b^ (% of control ligament) 3. Laxity ^ab^ (ratio of control ligament) (12 d)
Provenzano (2003) [46]	▪ 60 male rats (12 analyzed) ▪ BW (total sample): 245 ± 5 g (similar BW per group) ▪ Analgesic in water for 72 h postsurgery, consumed ad libitum. Similar access to food and water throughout the study. ▪ Knee MCL injury ▪ Surgical, transection bilaterally at the joint line	Hindlimb suspension	▪ Low: Ad libitum cage activity ▪ High: NA	▪ ~70° flexion ▪ Tensile to failure (10% strain/s)	1. Force at failure (N) ^b^ 2. Stress at failure (MPa) ^b^ 3. Stiffness (MPa) ^b^ 4. Strain at failure ^c^ (3, 7 wk)
Thornton (2003) [48]	▪ 53 female rabbits (40 analyzed) ▪ Age, 12 months ▪ Knee MCL injury ▪ Surgical, 4-mm midsubstance gap	Immobilisation (leg pinned at 150°-160° flexion)	▪ Low: Ad libitum cage activity ▪ High: NA	▪ 70° flexion ▪ Cyclic (2.2 MPa at wk 3 increasing to 7.1 MPa at wk 14; 30 cycles)	1. Laxity (mm)^d^ 2. N failures during creep testing (3, 6, and 14 wk)
Thornton (2005) [49]	▪ 64 female rabbits (22 analyzed) ▪ Knee MCL injury ▪ Surgical, 4-mm midsubstance gap	Immobilisation (leg pinned at 150°-160° flexion)	▪ Low: Ad libitum cage activity ▪ High: NA	▪ 70° flexion ▪ Cyclic (0.68 mm; 10 mm/min; 30 cycles); Tensile (0.68 mm; 20-min duration); Tensile to failure (20 mm/min)	1. Force at failure (N) 2. Stiffness (N/mm-2) 3. Laxity (Deformation at failure (mm) 4. Failure energy (N/mm) (6 and 14 wk)

MCL, medial collateral ligament; BW, body weight

^a^ ligament elongation prior to tensile resistance

^b^ data extracted from graph

^c^ no data/qualitative

^d^ ligament elongation during − 0.1 and + 0.1 N compression/tensile loading

^e^ data extracted for immobilisation vs. 5 min swim (no differences reported for 5 min vs. 15 min)

### Risk of bias in studies

All studies had at least one domain judged at a high risk of bias (Table 4). None of the studies provided sufficient details on sequence generation or allocation concealment. We considered body weight an important characteristic influencing treatment outcome; only two studies [46, 50] provided information on (similar) body weights at baseline. Other studies provided either no bodyweight data or only for the total study sample. Provenzano et al. [46] was the only study to give sufficient details on housing conditions, such as temperature and the duration of light-and-dark cycles. A lack of blinding of investigators and caregivers risked introducing bias in the five studies where comparisons involved free movement versus immobilization [47–49] or hindlimb suspension, [46] or where follow-up surgery was not matched with sham surgery in the control group [52].

**Table 4:** SYRCLE risk-of-bias assessment

	SYRCLE Criteria*									
Lead Author (Year)	1	2	3	4	5	6	7	8	9	10
Bray (1992) [47]	?	?	?	?	↑	?	↓	↓	?	↑
Burroughs (1990) [50]	?	↓	?	?	?	?	↓	↓	?	↓
Gomez (1991) [52]	?	?	?	?	↑	?	↓	↓	?	↓
Lechner (1991) [51]	?	?	?	?	?	?	↓	↓	?	↑
Provenzano (2003) [46]	?	↓	?	↓	↑	?	↓	↓	?	↓
Thornton (2003) [48]	?	?	?	?	↑	?	↓	↑	?	↓
Thornton (2005) [49]	?	?	?	?	↑	↑	↓	↑	↑	↑

↓ = low risk; ↑ = high risk; ? = unclear risk

*SYRCLE criteria: 1 = Sequence Generation; 2 = Baseline characteristics; 3 = Allocation concealment; 4 = Random housing; 5 = Blinding (investigators/caregivers); 6 = Random outcome assessment; 7 = Blinding (outcome assessors); 8 = Incomplete outcome data; 9 = Selective outcome reporting; 10 = Other sources of bias

Details on the use of random outcome assessment were unclear. One study limited the number of follow-ups within the control group due to earlier observations [49]. As all studies used automated tests with high precision and followed standardised procedures, we considered non-blinding of outcome assessors to be unlikely to affect the measurement readings. Bias due to incomplete outcome data was present due to specimen losses disproportionately affecting immobilised ligaments [48, 49]. Selective outcome reporting was unclear, as none of the studies referred to a prespecified study protocol. We note that all studies were carried out before the inception of reporting guidelines and registries specifically for animal research [53].

Other sources of bias included deviations from intended interventions [47–49] and unit of analysis errors [51]. Bray et al. [47] defined successful immobilisation as no voluntary joint motion and less than a few degrees of passive motion. However, even immediately following implementation, none of the animals met these criteria. At later healing intervals, passive knee range of motion was further increased. Two more studies used the same immobilisation protocol with no mention of remedial measures and are, therefore, also at high risk of bias [48, 49]. Lechner et al. [51] summarized data using the ratio between tested and contralateral (healthy) ligaments, prior to making between-group comparisons. Such an approach may introduce bias, as it assumes healthy ligament biomechanics were affected proportionally across treatment allocations.

Risk of bias judgments with supporting quotes from the original reports are available in Supplementary File 2.

### Study comparisons

#### Unloading vs. low load (4 studies)

**Fig. 2:**
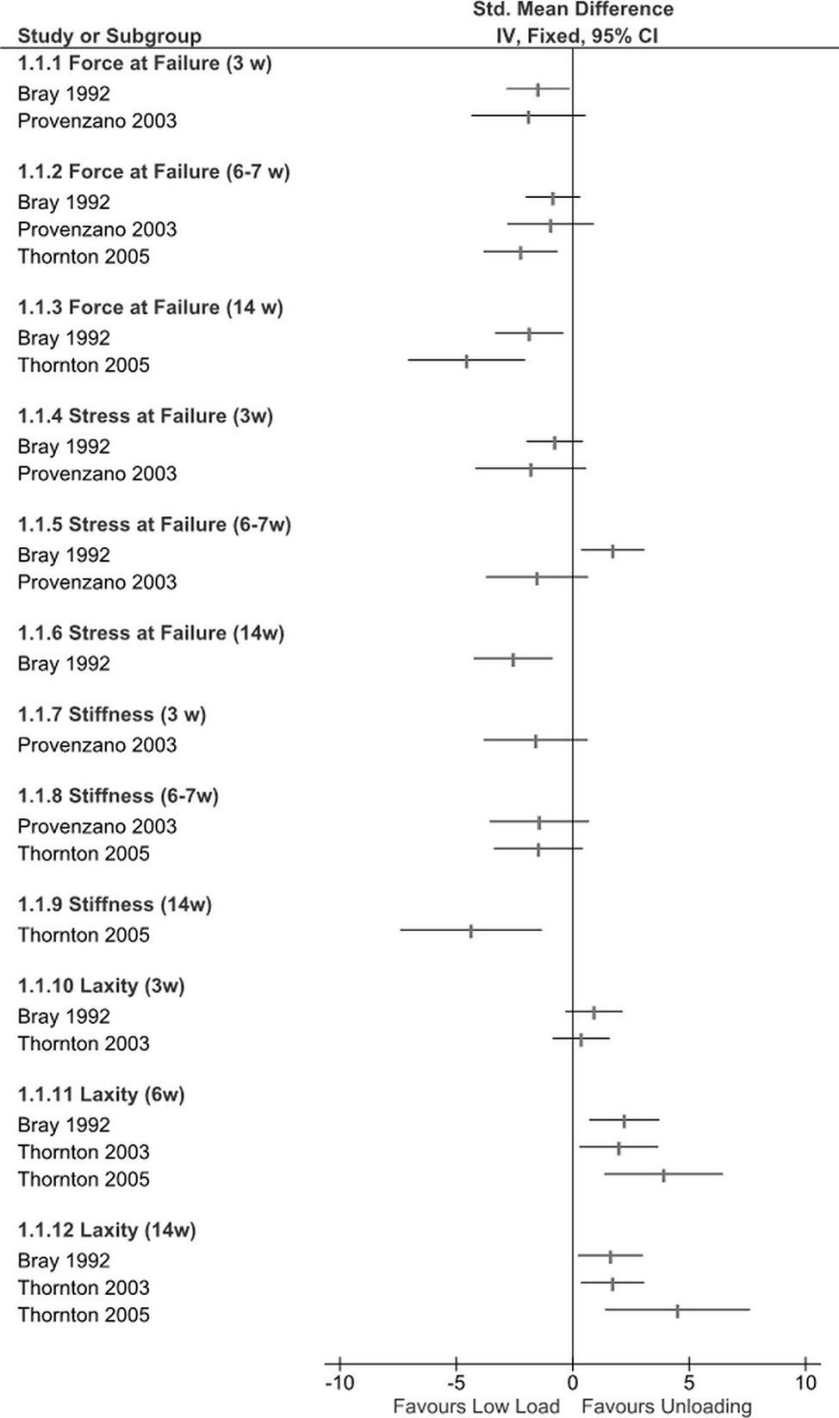
Forest Plot: Unloading vs. Low Load

Four studies compared limb unloading with a lower load strategy based on free (ad libitum) cage activity after an acute injury to the MCL [46–49]. Fig. 2 illustrates the magnitude and direction of effects for between-group comparisons across four outcomes related to ligament biomechanics: force at failure, stress at failure, stiffness, and laxity.

Three studies examined ligament force at failure [46, 47, 49]. There were trends favouring loading across all studies. Effects estimates were consistently large at early follow-ups (3 to 7 weeks post-injury) (Fig. 2); a pattern that continued to 14 weeks, where both Bray (SMD 1.9; 95% CI 0.4 to 3.3) [47] and Thornton (SMD 4.6; 95% CI 2.1 to 7.1) [49] found clear effects in favour of loading. Ligament stress at failure was examined by two studies. One of the studies found conflicting evidence over three follow-ups, [47] whereas the other recorded large effects in favour of loading at 3 and 6 weeks post injury [46]. Three studies examined ligament stiffness [46, 47, 49]. The ligaments in the loaded groups had greater stiffness than the ligaments in the unloaded groups. These results were consistent across all three studies and all follow-up periods. Although Bray et al. [47] only permitted a qualitative comparison of the force-deformation curves; the others had large effects in favour of loading at 7 weeks post injury (SMD 1.4; 95% CI − 0.7 to 3.6) [46] (SMD 1.5; 95% CI 0.4 to 3.4), [49] with the largest effect recorded at 14 weeks (SMD 4.4; 95% CI 1.3 to 7.4) [49].

Three studies [47–49] consistently reported greater laxity in loaded ligament groups. The largest between groups differences were reported by Thornton et al. [49] at both week 6 (SMD 3.9; 95% CI 1.4 to 6.4) and 14 (SMD 4.5; 95% CI 1.4 to 7.6) follow-ups. Tertiary outcomes not presented in Fig. 2 are number of failure events, ligament strain and energy at failure. The unloaded groups had more failure events during cyclic creep testing at 6 (RR 15.8; 95% CI 1.0 to 238) and 14 weeks post injury (RR 12.6; 95% CI 0.8 to 189); [48] with trends that loaded ligaments had a higher failure energy at 6 weeks (SMD 1.5; 95% CI − 0.41 to 3.4) and 14 weeks (SMD 2.7; 95% CI 0.5 to 4.8) [49]. One study [46] found strain at failure during tensile loading to be similar across treatment groups, but there were insufficient data in their manuscript to calculate an effect size.

#### Low load vs. high load (3 studies)

**Fig. 3:**
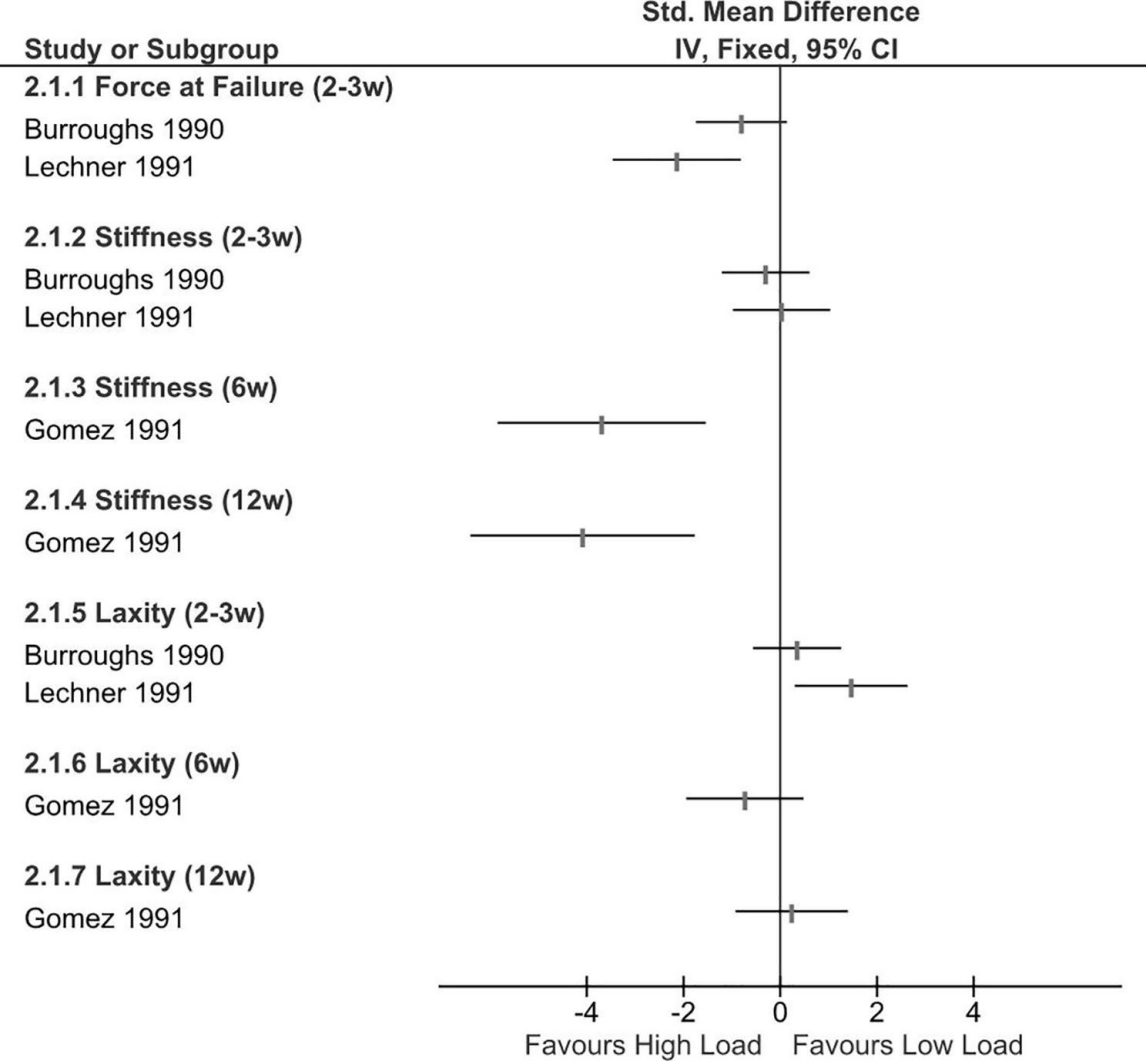
Forest Plot: Low Load vs. High Load

Figure 3 summarises the magnitude and direction of effects for between-group comparisons (low load vs. high load) [50–52] across three outcomes: force at failure, stiffness, and laxity. All three studies used free cage activity as their control (lower load) group, with two [50, 51] examining the additive effects of a formal exercise regimen. Burroughs et al. [50] had an exercise group undertaking five minutes of continuous swimming per day; and Lechner et al. [51] had their exercise group swim for ten minutes, rest for five, then repeat the swimming bout once again for a total of twenty minutes of exercise per day. In the third study [52] the high load intervention involved inducing constant tensile stress on the healing ligament from week 4 onwards (follow-up was at week 6 and week 12). The constant stress was caused by placing an intraarticular pin in situ, with controls undergoing sham surgery of the knee during the same day.

At two weeks post-injury, there were large effects in favour of higher load interventions (ad libitum activity plus structured exercise) for force at failure [50, 51]. Three studies assessed stiffness; [50–52] in two, [50, 51] there were no between group differences at two weeks post injury. However, Gomez el al. [52] reported large differences in stiffness in favour of the higher load group at six (SMD 3.7; 95% CI 1.6 to 5.8) and twelve weeks (SMD 4.1; 95% CI 1.8 to 6.4) post-injury. This study [52] also reported greater average stresses in the higher load group at 6 (SMD 1.9; 95% CI 0.4 to 3.3) and 12 (SMD 1.0; 95% CI − 0.2 to 2.3) weeks. The effects of higher loads on ligament laxity are unclear. At two weeks post-injury, trends suggest that higher loading results in greater (less advantageous) levels of ligament laxity [50, 51]. However, contradictory, and uncertain estimates were found at six and twelve-week follow-ups [52].

Burroughs et al. [50] also compared the effects of different durations of exercise (five vs. fifteen minutes of swimming per day), but found these conditions to be comparable in terms of ligament force at failure (SMD 0.1; 95% CI − 0.8 to 1.0), stiffness (SMD 0.05; 95% CI − 0.8 to 0.9), and laxity (SMD 0.02; 95% CI − 0.9 to 0.9).

## Discussion

Clinical studies suggest that progressive exercise is more effective than immobilisation after common lower limb ligament injury (e.g. ankle sprain) [11–13]. These effects may be underpinned by mechanotransduction, whereby tissue loading stimulates healing, optimising tissue mass, and ultimately restoring ligament biomechanics. This is difficult to corroborate within clinical research, as key biomechanical constructs such as ligament stiffness or maximum strength cannot be quantified. This is the first systematic review examining the biomechanical effects of early loading on an injured ligament. Seven controlled animal trials were included: all assessing the knee MCL. All studies were at a high risk of bias and thus limiting the extent of our conclusions.

This review found preliminary evidence that ad libitum loading post-injury increases both ligament force at failure and ligament stiffness, compared to unloading interventions (immobilisation; hindlimb suspension). Exposing healthy ligaments to sub failure tension loading, typically results in increased strength, size, improvements in matrix organization, and collagen content [54]. Data from injured animal models consistently show that when healing ligaments are load deprived, they are histologically inferior and present with increased cellularity; [52] less mature tissue development at the injury site; [55] extracellular matrix discontinuity and collagen misalignment [52] [41] [46] (compared to loaded tissue). The current review emphasizes the importance of re-introducing load after injury, to optimise tissue mechanics. The positive biomechanical effects associated with tissue loading, are underpinned by mechanotransduction, whereby induced loads strain the ligament cells, which then convert the tensile mechanical stimuli into cellular and molecular response that promote structural changes [15, 56]. This has been validated through gene expression analysis in animal models, where post-injury loading upregulated synthesis of new collagen and other key constituents of the extracellular matrix (e.g. biglycan, matrix metalloproteinase [MMP]–2, and tissue inhibitor matrix metalloproteinase [TIMP]–1) (compared to stress shielding) [39]. Others [57] demonstrate that mechanically loading ligaments, up-regulates expression of key collagen-modifying enzymes (lysyl oxidase; lysyl hydroxylases), with corresponding increases in collagen cross-linking.

Our review also suggests that ligaments adapt to loading slowly. Although there were consistent effects in favour of early loading (vs. unloading), the most precise estimates (corresponding 95% CIs excluding zero) were recorded at the longest follow up time points (12–14 weeks post-injury). Data from two studies [47, 48] suggest that tissue mechanics remain below pre-injury levels after 12 weeks of loading, with ligament strength values at just 40% of uninjured controls. Canine models [29] also show that the mechanical properties of the MCL substance have not fully recovered by 48 weeks post injury. The average time to return to sport after an ankle ligament injury or knee MCL injury is approximately two [4, 58] to three [3, 7] weeks respectively; this recovery period may be inadequate for complete restoration of ligament mechanics and could explain the high prevalence of reinjury [3, 10].

Treatment effects were not consistent across outcomes, and loading had a detrimental impact on ligament laxity. Since most studies measured laxity at very low tensile loads, the data characterise ligament behaviour in the nonlinear, toeing region of the stress/strain curve. Stiffness and strength data were recorded under higher loading conditions and therefore reflect tissue behaviour in the linear and yielding/breaking regions of the curve. We note that loading interventions were consistently initiated in the immediate stages post-injury. This exposes the ligament to a repetitive constant load, potentially promoting excessive creep behaviour and long-term detriments to nonlinear stress/strain behaviour. The clinical implications of higher laxity behaviour (in response to early loading following injury) are unclear. Ligaments are rich in mechanoreceptors which mediate important afferent signals when the tissue is loaded; this optimises joint stability and synergistic muscle activity [59]. Clinical studies suggest patients with ankle instability have reduced mechanoreceptor function in response to low amplitude ligament loading compared to healthy controls, [60] but this may not translate to higher loading environments. Current clinical recommendations for ligament rehabilitation include a short period of protection followed by progressive loading; [61, 62] future studies should ascertain if such an approach optimises both nonlinear and linear stress/strain behaviour.

An optimal loading protocol did not emerge, as few included studies manipulated loading parameters (e.g. type, frequency). A potentially interesting finding [50] was that increasing a loading bout from 5 to 15 min per day had minimal effect on biomechanical outcomes. This aligns with evidence derived from related tissue models. Baar et al. [63] found that engineered ligaments were most responsive to short periods of loading (< 10 min), after which a refractory period ensued (of ~ 6 h), where tissues were unresponsive to load. Healthy bone [64] and injured tendon models also seem to respond optimally to shorter loading bouts, interspersed with prolonged rest periods. It may be that the mechanical sensitivity of connective tissue may saturate quickly; one author [65] succinctly suggests that tissues “remember” the stimulation from a single loading episode for most of the day. The magnitude of load may also moderate treatment outcome; [16] but between-group differences for lower (ad libitum loading) vs. higher load interventions (ad libitum plus additional exercise) were more inconsistent across outcomes and often had overlapping confidence intervals. Ligaments may be most responsive to tensile, cyclical loading, achieved over various joint positions and ranges [16]. It is possible that the ‘higher loading’ strategies employed in the included studies (based on swimming or passive tensile forces) were suboptimal and induced insufficient mechanical stimulation.

### Limitations and future research

All included studies were designed and conducted before the publication of the SYRCLE risk-of-bias criteria [18]. This may explain the overall high risk of bias reported. Most studies incorporated a small number of animal models, possibly contributing to the wide and non-significant confidence intervals reported. These factors limit the validity of our findings, and we can only make preliminary conclusions. Future research in this field must be undertaken in line with SYRCLE criteria and use adequate sample sizes.

We had originally planned to conduct a meta-analysis. This was not undertaken due to the considerable clinical heterogeneity across the included studies, relating to injury severity (isolated vs. combined MCL injury) and differences in the nature of the immobilisation and exercise interventions. These factors likely influenced the biomechanical outcomes in the included studies. Similarly, there were insufficient data for our planned subgroup analysis on treatment intervention (low dose loading vs. higher dose loading). Since all studies scored poorly on our risk of bias assessment, we could not undertake a preplanned sensitivity analysis (that would have excluded studies at a high risk of bias).

All included studies used rat or rabbit models and we acknowledge the differences in the timelines for adequate repair after ligament injury compared to humans. The injury models performed a complete transection at the mid-portion of the knee MCL. This does not fully align with clinical presentation, since humans often suffer more fragmented injuries to the distal or proximal thirds of the ligament [3]. All studies tested ligament biomechanics until failure, but this was induced using a range of loading magnitudes and strain rates. Test protocols varied from cyclic loading between 0 and 4% strain, to tensile loading at a strain rate of 0.25 to 0.33 mm/second. By contrast, the mean failure strain for human knee ligaments (ACL, MCL) is estimated at 15%, [66, 67] occurring within 50 ms of ground contact [68].

Nonetheless, the data in our review still provide important insights into ligament healing that are not feasible in human research due to ethical considerations. Because of the small number of included studies, we are limited to preliminary conclusions for our second objective; determining if loading parameters moderate biomechanical outcomes after injury. Future animal models should incorporate follow-ups beyond 14 weeks and prioritize study designs that manipulate the nature, magnitude, duration, and frequency of loading.

The most recent study included in the current review was published in 2005. This may reflect temporal trends in human ligament research, with an increased focus on surgical management, orthobiologic therapies, [69] and functional tissue engineering [70]. These innovations are important, but a large proportion of ligament injuries will continue to be managed conservatively. There are currently only two human studies [71, 72] supporting the idea that human ligaments hypertrophy in response to mechanical loading. These studies used a cross-sectional design on healthy subjects, and ligament strength was inferred from the cross-sectional area on MRI assessment. Although animal models are less ecologically valid, they still provide the best medium for quantifying ligaments’ material properties post-injury (e.g. ultimate tensile strength, stiffness) and post-loading. Therefore, developing a robust understanding of the biomechanical effects of ligament loading and any parameter/dose-dependent effects requires a combination of well-designed animal models and randomised clinical trials.

## Conclusion

Undertaking ad libitum loading after a lower limb ligament injury generates stronger and stiffer ligament tissue than unloading or immobilisation. These tissue adaptations are slow, with large and precise between-group effects only evident at 14 weeks post-injury. Ad libitum loading may have a detrimental effect on low load tissue behaviour, based on greater laxity during initial ligament recruitment. The optimal stimulus for healing ligaments remains unclear. Future research must examine the impact of manipulating key loading parameters (e.g. nature, magnitude, duration, frequency of loading) using sufficiently sized study groups. All findings are preliminary due to the high risk of bias of included studies and statistical imprecision surrounding the outcome measures.

## Electronic supplementary material

Below is the link to the electronic supplementary material.

Supplementary Material 1

Supplementary Material 2

## Data Availability

Our RevMan file with complete information on primary, secondary and tertiary outcomes is available in an open access data repository: https://doi.org/10.5281/zenodo.5607370.

## References

[1] Ornon G, Ziltener JL, Fritschy D, Menetrey J (2020). Epidemiology of injuries in professional ice hockey: a prospective study over seven years. J Exp Orthop.

[2] Mack CD, Kent RW, Coughlin MJ, Shiue KY, Weiss LJ, Jastifer JR, Wojtys EM, Anderson RB (2020). Incidence of Lower Extremity Injury in the National Football League: 2015 to 2018. Am J Sports Med.

[3] Lundblad M, Walden M, Magnusson H, Karlsson J, Ekstrand J (2013). The UEFA injury study: 11-year data concerning 346 MCL injuries and time to return to play. Br J Sports Med.

[4] Walden M, Hagglund M, Ekstrand J (2013). Time-trends and circumstances surrounding ankle injuries in men’s professional football: an 11-year follow-up of the UEFA Champions League injury study. Br J Sports Med.

[5] Lopez V, Ma R, Weinstein MG, Hume PA, Cantu RC, Victoria C, Queler SC, Webb KJA, Allen AA (2020). United States Under-19 Rugby-7s: incidence and nature of Match Injuries during a 5-year Epidemiological Study. Sports Med Open.

[6] West SW, Starling L, Kemp S, Williams S, Cross M, Taylor A, Brooks JHM, Stokes KA (2021). Trends in match injury risk in professional male rugby union: a 16-season review of 10 851 match injuries in the English premiership (2002–2019): the Professional Rugby Injury Surveillance Project. Br J Sports Med.

[7] Roach CJ, Haley CA, Cameron KL, Pallis M, Svoboda SJ, Owens BD (2014). The epidemiology of medial collateral ligament sprains in young athletes. Am J Sports Med.

[8] Doherty C, Delahunt E, Caulfield B, Hertel J, Ryan J, Bleakley C (2014). The incidence and prevalence of ankle sprain injury: a systematic review and meta-analysis of prospective epidemiological studies. Sports Med.

[9] Doherty C, Bleakley C, Hertel J, Caulfield B, Ryan J, Delahunt E (2016). Recovery from a first-time lateral ankle sprain and the predictors of chronic ankle instability: a prospective cohort analysis. Am J Sports Med.

[10] Kramer DE, Miller PE, Berrahou IK, Yen YM, Heyworth BE (2020). Collateral ligament knee injuries in Pediatric and adolescent athletes. J Pediatr Orthop.

[11] Bleakley CM, McDonough SM, MacAuley DC (2008). Some conservative strategies are effective when added to controlled mobilisation with external support after acute ankle sprain: a systematic review. Aust J Physiother.

[12] Bleakley CM, Taylor JB, Dischiavi SL, Doherty C, Delahunt E (2019). Rehabilitation exercises reduce Reinjury Post Ankle Sprain, but the content and parameters of an optimal Exercise Program have yet to be established: a systematic review and Meta-analysis. Arch Phys Med Rehabil.

[13] Doherty C, Bleakley C, Delahunt E, Holden S (2017). Treatment and prevention of acute and recurrent ankle sprain: an overview of systematic reviews with meta-analysis. Br J Sports Med.

[14] Miklovic TM, Donovan L, Protzuk OA, Kang MS, Feger MA (2018). Acute lateral ankle sprain to chronic ankle instability: a pathway of dysfunction. Phys Sportsmed.

[15] Thompson WR, Scott A, Loghmani MT, Ward SR, Warden SJ (2016). Understanding mechanobiology: physical therapists as a force in Mechanotherapy and Musculoskeletal Regenerative Rehabilitation. Phys Ther.

[16] Glasgow P, Phillips N, Bleakley C (2015). Optimal loading: key variables and mechanisms. Br J Sports Med.

[17] Page MJ, McKenzie JE, Bossuyt PM, Boutron I, Hoffmann TC, Mulrow CD, Shamseer L, Tetzlaff JM, Akl EA, Brennan SE, Chou R, Glanville J, Grimshaw JM, Hrobjartsson A, Lalu MM, Li T, Loder EW, Mayo-Wilson E, McDonald S, McGuinness LA, Stewart LA, Thomas J, Tricco AC, Welch VA, Whiting P, Moher D (2021). The PRISMA 2020 statement: an updated guideline for reporting systematic reviews. Int J Surg.

[18] Hooijmans CR, Rovers MM, de Vries RB, Leenaars M, Ritskes-Hoitinga M, Langendam MW (2014). SYRCLE’s risk of bias tool for animal studies. BMC Med Res Methodol.

[19] Cohen J. Statistical power analysis for the behavioural sciences. Routledge; 1998.

[20] Ng JWG, Myint Y, Ali FM (2020). Management of multiligament knee injuries. EFORT Open Rev.

[21] Thornton GM, Bailey SJ (2012). Repetitive loading damages healing ligaments more than sustained loading demonstrated by reduction in modulus and residual strength. J Biomech.

[22] Thornton GM, Bailey SJ (2013). Healing ligaments have shorter lifetime and greater strain rate during fatigue than creep at functional stresses. J Biomech Eng.

[23] Chamberlain CS, Brounts SH, Sterken DG, Rolnick KI, Baer GS, Vanderby R (2011). Gene profiling of the rat medial collateral ligament during early healing using microarray analysis. J Appl Physiol (1985).

[24] Inoue M, Woo SL, Gomez MA, Amiel D, Ohland KJ, Kitabayashi LR (1990). Effects of surgical treatment and immobilization on the healing of the medial collateral ligament: a long-term multidisciplinary study. Connect Tissue Res.

[25] Gomez MA, Woo SL, Inoue M, Amiel D, Harwood FL, Kitabayashi L (1989). Medical collateral ligament healing subsequent to different treatment regimens. J Appl Physiol (1985).

[26] Woo SL, Gomez MA, Inoue M, Akeson WH (1987). New experimental procedures to evaluate the biomechanical properties of healing canine medial collateral ligaments. J Orthop Res.

[27] Korkala O, Rusanen M, Gronblad M (1984). Healing of experimental ligament rupture: findings by scanning electron microscopy. Arch Orthop Trauma Surg.

[28] Frank C, Amiel D, Akeson WH (1983). Healing of the medial collateral ligament of the knee. A morphological and biochemical assessment in rabbits. Acta Orthop Scand.

[29] Woo SL, Inoue M, McGurk-Burleson E, Gomez MA (1987). Treatment of the medial collateral ligament injury. II: structure and function of canine knees in response to differing treatment regimens. Am J Sports Med.

[30] El Saman M, Hutzschenreuter P, Claes L. Tensile strength of partially cut lateral knee ligaments following, or without plaster immobilization. Chir Forum Exp Klin Forsch 1978, (1978)(1978):267–269. 752598

[31] Vailas AC, Tipton CM, Matthes RD, Gart M (1981). Physical activity and its influence on the repair process of medial collateral ligaments. Connect Tissue Res.

[32] Sakuma K, Mizuta H, Kai K, Takagi K, Iyama K (1993). Ultrastructural changes of collagen fibers in the anterior cruciate ligament of bipedal rats after enforced running. Nihon Seikeigeka Gakkai Zasshi.

[33] Boorman RS, Shrive NG, Frank CB (1998). Immobilization increases the vulnerability of rabbit medial collateral ligament autografts to creep. J Orthop Res.

[34] Wren TA, Beaupre GS, Carter DR (2000). Tendon and ligament adaptation to exercise, immobilization, and remobilization. J Rehabil Res Dev.

[35] Thornton GM, Boorman RS, Shrive NG, Frank CB (2002). Medial collateral ligament autografts have increased creep response for at least two years and early immobilization makes this worse. J Orthop Res.

[36] Demirhan M, Uysal M, Kilicoglu O, Atalar AC, Sivacioglu S, Solakoglu S, Bozdag E, Sunbuloglu E (2005). Tensile strength of ligaments after thermal shrinkage depending on time and immobilization: in vivo study in the rabbit. J Shoulder Elbow Surg.

[37] van Royen BJ, O’Driscoll SW, Dhert WJ, Salter RB (1986). A comparison of the effects of immobilization and continuous passive motion on surgical wound healing in mature rabbits. Plast Reconstr Surg.

[38] Kokubun T, Kanemura N, Murata K, Moriyama H, Morita S, Jinno T, Ihara H, Takayanagi K (2016). Effect of changing the joint kinematics of Knees with a ruptured anterior cruciate ligament on the molecular Biological responses and spontaneous Healing in a rat model. Am J Sports Med.

[39] Martinez DA, Vailas AC, Vanderby R, Grindeland RE (2007). Temporal extracellular matrix adaptations in ligament during wound healing and hindlimb unloading. Am J Physiol Regul Integr Comp Physiol.

[40] Hurschler C, Provenzano PP, Vanderby R (2003). Scanning electron microscopic characterization of healing and normal rat ligament microstructure under slack and loaded conditions. Connect Tissue Res.

[41] Padgett LR, Dahners LE (1992). Rigid immobilization alters matrix organization in the injured rat medial collateral ligament. J Orthop Res.

[42] Frank C, MacFarlane B, Edwards P, Rangayyan R, Liu ZQ, Walsh S, Bray R (1991). A quantitative analysis of matrix alignment in ligament scars: a comparison of movement versus immobilization in an immature rabbit model. J Orthop Res.

[43] Ozenci AM, Panjabi MM (2005). Injured rabbit ACL treated by radiofrequency. Effects of cyclic loading. Clin Biomech (Bristol Avon).

[44] Majima T, Marchuk LL, Sciore P, Shrive NG, Frank CB, Hart DA (2000). Compressive compared with tensile loading of medial collateral ligament scar in vitro uniquely influences mRNA levels for aggrecan, collagen type II, and collagenase. J Orthop Res.

[45] Hart DP, Dahners LE (1987). Healing of the medial collateral ligament in rats. The effects of repair, motion, and secondary stabilizing ligaments. J Bone Joint Surg Am.

[46] Provenzano PP, Martinez DA, Grindeland RE, Dwyer KW, Turner J, Vailas AC, Vanderby R (2003). Hindlimb unloading alters ligament healing. J Appl Physiol (1985).

[47] Bray RC, Shrive NG, Frank CB, Chimich DD (1992). The early effects of joint immobilization on medial collateral ligament healing in an ACL-deficient knee: a gross anatomic and biomechanical investigation in the adult rabbit model. J Orthop Res.

[48] Thornton GM, Shrive NG, Frank CB (2003). Healing ligaments have decreased cyclic modulus compared to normal ligaments and immobilization further compromises healing ligament response to cyclic loading. J Orthop Res.

[49] Thornton GM, Johnson JC, Maser RV, Marchuk LL, Shrive NG, Frank CB (2005). Strength of medial structures of the knee joint are decreased by isolated injury to the medial collateral ligament and subsequent joint immobilization. J Orthop Res.

[50] Burroughs P, Dahners LE (1990). The effect of enforced exercise on the healing of ligament injuries. Am J Sports Med.

[51] Lechner CT, Dahners LE (1991). Healing of the medial collateral ligament in unstable rat knees. Am J Sports Med.

[52] Gomez MA, Woo SL, Amiel D, Harwood F, Kitabayashi L, Matyas JR (1991). The effects of increased tension on healing medical collateral ligaments. Am J Sports Med.

[53] Percie du Sert N, Hurst V, Ahluwalia A, Alam S, Avey MT, Baker M, Browne WJ, Clark A, Cuthill IC, Dirnagl U, Emerson M, Garner P, Holgate ST, Howells DW, Karp NA, Lazic SE, Lidster K, MacCallum CJ, Macleod M, Pearl EJ, Petersen OH, Rawle F, Reynolds P, Rooney K, Sena ES, Silberberg SD, Steckler T, Wurbel H (2020). The ARRIVE guidelines 2.0: updated guidelines for reporting animal research. BMJ Open Sci.

[54] Logerstedt DS, Ebert JR, MacLeod TD, Heiderscheit BC, Gabbett TJ, Eckenrode BJ (2022). Effects of and response to mechanical loading on the knee. Sports Med.

[55] Goldstein WM, Barmada R (1984). Early mobilization of rabbit medial collateral ligament repairs: biomechanic and histologic study. Arch Phys Med Rehabil.

[56] Khan KM, Scott A (2009). Mechanotherapy: how physical therapists’ prescription of exercise promotes tissue repair. Br J Sports Med.

[57] Kaku M, Rosales Rocabado JM, Kitami M, Ida T, Akiba Y, Yamauchi M, Uoshima K (2016). Mechanical loading stimulates expression of collagen cross-linking Associated enzymes in Periodontal Ligament. J Cell Physiol.

[58] Kofotolis ND, Kellis E, Vlachopoulos SP (2007). Ankle sprain injuries and risk factors in amateur soccer players during a 2-year period. Am J Sports Med.

[59] Solomonow M, Krogsgaard M (2001). Sensorimotor control of knee stability. A review. Scand J Med Sci Sports.

[60] Needle AR, Charles B, Buz S, Farquhar WB, Thomas SJ, Rose WC, Kaminski TW (2013). Muscle spindle traffic in functionally unstable ankles during ligamentous stress. J Athl Train.

[61] Bleakley CM, Glasgow P, MacAuley DC (2012). PRICE needs updating, should we call the POLICE?. Br J Sports Med.

[62] Kaminski TW, Hertel J, Amendola N, Docherty CL, Dolan MG, Hopkins JT, Nussbaum E, Poppy W, Richie D (2013). National athletic Trainers’ Association: national athletic Trainers’ Association position statement: conservative management and prevention of ankle sprains in athletes. J Athl Train.

[63] Baar K (2017). Minimizing Injury and Maximizing Return to play: Lessons from Engineered ligaments. Sports Med.

[64] Burr DB, Robling AG, Turner CH (2002). Effects of biomechanical stress on bones in animals. Bone.

[65] Andersson T, Eliasson P, Aspenberg P (2009). Tissue memory in healing tendons: short loading episodes stimulate healing. J Appl Physiol (1985).

[66] Bates NA, Schilaty ND, Nagelli CV, Krych AJ, Hewett TE (2018). Validation of Noncontact Anterior Cruciate ligament tears produced by a mechanical impact Simulator against the clinical presentation of Injury. Am J Sports Med.

[67] Woo SL, Hollis JM, Adams DJ, Lyon RM, Takai S (1991). Tensile properties of the human femur-anterior cruciate ligament-tibia complex. The effects of specimen age and orientation. Am J Sports Med.

[68] Krosshaug T, Nakamae A, Boden BP, Engebretsen L, Smith G, Slauterbeck JR, Hewett TE, Bahr R (2007). Mechanisms of anterior cruciate ligament injury in basketball: video analysis of 39 cases. Am J Sports Med.

[69] Lin KM, Frey CS, Atzmon R, Pierre K, Vel MS, Sherman SL (2023). Orthobiologic techniques for Surgical Augmentation. Phys Med Rehabil Clin N Am.

[70] Butler DL (2022). Evolution of functional tissue engineering for tendon and ligament repair. J Tissue Eng Regen Med.

[71] Beaulieu ML, DeClercq MG, Rietberg NT, Li SH, Harker EC, Weber AE, Ashton-Miller JA, Wojtys EM (2021). The anterior cruciate ligament can become hypertrophied in response to mechanical loading: a magnetic resonance imaging study in Elite athletes. Am J Sports Med.

[72] Grzelak P, Podgorski M, Stefanczyk L, Krochmalski M, Domzalski M (2012). Hypertrophied cruciate ligament in high performance weightlifters observed in magnetic resonance imaging. Int Orthop.

