# Supplementary material for: Does mechanical loading restore ligament biomechanics after injury? A systematic review of studies using animal models
Source: BMC Musculoskelet Disord. 2023 Jun 22;24:511. doi: 10.1186/s12891-023-06653-x (PMC10286351; doi:10.1186/s12891-023-06653-x)
Supplement: Supplementary file 1 — Supplementary Material 1 [file 12891_2023_6653_MOESM1_ESM.docx]

**SUPPLEMENTAL FILE 1 - DETAILED SEARCH INFORMATION**

**ELECTRONIC DATABASE SEARCH STRATEGIES**

**Database:** Ovid MEDLINE® ALL <1946 to May 29, 2023>

**Search Executed:** May 29, 2023 (date of updated search)

| Sequence | Search term(s) used | Number of hits generated |
| --- | --- | --- |
| 1 | (protection or rest or functional or movement or exercise or loading or unloading or weight-bearing or weight-bear or cast or splint or immobilisation or immobilization).mp. | 3,137,638 |
| 2 | exp Ligaments/ | 45,070 |
| 3 | exp "Sprains and Strains"/ | 20,625 |
| **4** | **2 or 3** | **64,354** |
| **5** | **1 and 4** | **14,286** |
| **6** | **Limit 5 to animals** | **1,719** |

**Database:** Embase <1974 to 2023 Week 21>

**Search Executed:** May 29, 2023 (date of updated search)

| Sequence | Search term(s) used | Number of hits generated |
| --- | --- | --- |
| 1 | (protection or rest or functional or movement or exercise or loading or unloading or weight-bearing or weight-bear or cast or splint or immobilisation or immobilization).mp. | 7,292,320 |
| 2 | ligament/ | 38,720 |
| 3 | sprain/ | 8,550 |
| **4** | **2 or 3** | **47,052** |
| **5** | **1 and 4** | **9,487** |
| **6** | **Limit 5 to animals and animal studies** | **702** |

**Database:** Agricultural & Environmental Sciences Collection <1960 to 2023 Week 21>

**Search Executed:** May 29, 2023 (date of updated search)

| Sequence | Search term(s) used | Number of hits generated |
| --- | --- | --- |
| 1 | Ligament | 47,865 |
| 2 | Controlled trial | 594,588 |
| **3** | **1 and 2** | **8,418** |
| **4** | **Limit 3 to animals** | **384** |

The lead author developed all the primary electronic database search strategies.

**SUPPLEMENTARY SEARCH STRATEGIES**

PubMed’s “search all similar articles” function (May 12, 2021), “cited by” function (May 29, 2023), and hand searching of all studies meeting the inclusion criteria:

| Author(s) and year of publication | PMID | Number of references hand searched (backward citation tracking) | Number of articles screened that cited included studies  (forward citation tracking) | Number of similar articles screened |
| --- | --- | --- | --- | --- |
| **Eligible studies identified through primary searches** | | | |  |
| Bray et al. 1992 | 1740733 | 16 | 13 | 546 |
| Burroughs et al. 1990 | 2403185 | 9 | 11 | 116 |
| Lechner et al. 1991 | 1962719 | 16 | 4 | 100 |
| Provenzano et al. 2003 | 12391134 | 59 | 21 | 108 |
| Thornton et al. 2003 | 12798073 | 19 | 15 | 200 |
| Thornton et al. 2005 | 16140200 | 22 | 7 | 225 |
| **Eligible studies identified through supplementary searches** | | | |  |
| Gomez et al. 1991 | 1897648 | 28 | 15 | 171 |

For additional context regarding each step of the eligibility screening process, please see the flow chart in Figure 1 of the manuscript.
