# Supplementary material for: Does mechanical loading restore ligament biomechanics after injury? A systematic review of studies using animal models
Source: BMC Musculoskelet Disord. 2023 Jun 22;24:511. doi: 10.1186/s12891-023-06653-x (PMC10286351; doi:10.1186/s12891-023-06653-x)
Supplement: Supplementary file 2 — Supplementary Material 2 [file 12891_2023_6653_MOESM2_ESM.docx]

| **Supplemental file 2.** SYRCLE judgments with quotes and comments | |
| --- | --- |
| **Bias in sequence generation** | |
| Bray et al. 1992 | **Unclear**  There is no mention of how the animals were selected for the different study arms. |
| Burroughs et al. 1990 | **Unclear**  There is no mention of how the animals were selected for the different study arms. |
| Gomez et al. 1991 | **Unclear**  There is no mention of how the animals were selected for the different study arms.  *“Twenty-four New Zealand White rabbits… were divided into four groups with six animals in each group.”* |
| Lechner et al. 1991 | **Unclear**  There is no mention of how the animals were selected for the different study arms.  *“Forty male Sprague-Dawley rats… were divided into four treatment groups”* |
| Provenzano et al. 2003 | **Unclear**  Although randomization is mentioned, it is not stated how or by whom it was performed.  *“Each time group was composed of three subgroups, each containing 10 randomly selected animals…”* |
| Thornton et al. 2003 | **Unclear**  There is no mention of how the animals were selected for the different study arms.  ***“****Fifty-three female one-year-old New Zealand White rabbits were allocated to two surgical groups (unilateral MCL gap surgery with n = 23 and bilateral MCL gap surgery with n = 17) and one normal group (n = 13).”* |
| Thornton et al. 2005 | **Unclear**  There is no mention of how the animals were selected for the different study arms.  *“Fifty-two animals underwent surgery…Twenty-nine of these animals had their right hindlimbs pin immobilized in full flexion while left (contralateral to immobilized) hindlimbs were not immobilized. The remaining 23 surgical animals had no immobilization of either hindlimb.”* |
| **Bias in baseline characteristics** | |
| Bray et al. 1992 | **Unclear**  Information is given on type of animal, sex, and bodyweight. However, bodyweight is only given per the total sample and not by study arm.  *“Forty-six adult, female, New Zealand white rabbits (aged 12 months) weighing between 3.6 and 6.4 kg (mean of 4.7±0.66 kg) were used”* |
| Burroughs et al. 1990 | **Low risk**  Information is given on type of animal, sex, and (similar) bodyweights per group.  *“Fifty large male Sprague-Dawley rats… treatment groups had similar mean weights at the beginning of the experiment. The weights were: Group A, 625 ± 68 g; Group B, 624 ± 65 g; Group C, 626 ± 57 g; Group D, 603 ± 52 g; and Group E, 608 ± 58 g”* |
| Gomez et al. 1991 | **Unclear**  Information is given on type of animal, sex, and bodyweight. However, bodyweight is only given per the total sample and not by study arm.  *“Twenty-four New Zealand White rabbits, weighing 3.3 ± 0.11 kg, were divided into four groups with six animals in each group.”* |
| Lechner et al. 1991 | **Unclear**  Information is given on type of animal, sex, and bodyweight. However, bodyweight is only given per the total sample and not by study arm.  *“Forty male Sprague-Dawley rats (weight, 400 to 600 g) were divided into four treatment groups”* |
| Provenzano et al. 2003 | **Low risk**  Information is given on type of animal, sex, and (similar) bodyweights per group.  *“Sixty male Sprague-Dawley rats (245±5 g) were used as an*  *animal model… Each time group was composed of three subgroups, each containing 10 randomly selected animals… Initial body weights were carefully selected so that the groups were not different.”* |
| Thornton et al. 2003 | **Unclear**  Information is given on type of animal, age, and sex – but there is no mention of bodyweight at baseline.  *“Fifty-three female one-year-old New Zealand White rabbits were*  *allocated to two surgical groups (unilateral MCL gap surgery with*  *n = 23 and bilateral MCL gap surgery with n = 17) and one normal*  *group (n = 13).”* |
| Thornton et al. 2005 | **Unclear**  Information is only given on type of animal and sex.  *“Sixty-four female New Zealand White rabbits were used in this study….”* |
| **Bias in allocation concealment** | |
| Bray et al. 1992 | **Unclear**  There is no mention of allocation strategy. |
| Burroughs et al. 1990 | **Unclear**  There is no mention of allocation strategy. |
| Gomez et al. 1991 | **Unclear**  There is no mention of allocation strategy. |
| Lechner et al. 1991 | **Unclear**  There is no mention of allocation strategy. |
| Provenzano et al. 2003 | **Unclear**  Although randomization is mentioned, it is not stated how or by whom it was performed.  *“Each time group was composed of three subgroups, each containing 10 randomly selected animals…”* |
| Thornton et al. 2003 | **Unclear**  There is no mention of allocation strategy. |
| Thornton et al. 2005 | **Unclear**  There is no mention of allocation strategy. |
| **Bias in housing conditions** | |
| Bray et al. 1992 | **Unclear**  There is no mention of housing conditions besides nutrition,  *“All animals received similar diets postoperatively… and water ad libitum.”* |
| Burroughs et al. 1990 | **Unclear**  There is no mention of housing conditions. |
| Gomez et al. 1991 | **Unclear**  Cage dimensions are mentioned, but not placement or temperature.  *“…animals were allowed… weeks of healing time with free cage activity (cage dimensions, 1.0 X 0.6 X 1.0 m).”* |
| Lechner et al. 1991 | **Unclear**  Cage dimensions are mentioned, but not placement or temperature.  *“The rats… were allowed unrestricted cage activity (cage dimensions, 20 X 30 cm) following surgery…”* |
| Provenzano et al. 2003 | **Low risk**  Housing conditions are sufficiently detailed to determine that they are similar.  *“All animals were given an analgesic (Tylenol/codeine) in*  *their water for 72 h postsurgery….* *All animals were under a 12:12-h light-dark cycle in a room at 24°C. Rats were fed Purina rat chow, watered ad libitum, and checked twice daily for overall health, skin incision healing, food and water consumption, and the condition of their tails (the harness should prevent slippage without restricting circulation).”* |
| Thornton et al. 2003 | **Unclear**  There is no mention of housing conditions. |
| Thornton et al. 2005 | **Unclear**  There is no mention of housing conditions. |
| **Bias in blinding of caregivers and investigators** | |
| Bray et al. 1992 | **High risk**  There are visually discernible differences between the treatment groups.  *“animals… had their right knee immobilized at approximately 150-160” of flexion by means of an extra-articular 1.6 mm stainless steel transfixing pin”* |
| Burroughs et al. 1990 | **Unclear**  Although surgical differences were not visually discernible, there is insufficient information to warrant a low risk of bias. |
| Gomez et al. 1991 | **High risk**  There are visually discernible differences between the treatment groups, as the increased tension groups were not matched with sham surgery in the ad-lib movement groups at the second surgery.  *“After… weeks of cage activity, the animal was again operated on; a stainless steel pin, 9.5 mm long and 1.6 mm in diameter, was placed perpendicularly underneath the healing MCL, distal to the joint line.”* |
| Lechner et al. 1991 | **Unclear**  Although surgical differences were not visually discernible, there is insufficient information to warrant a low risk of bias. |
| Provenzano et al. 2003 | **High risk**  There are visually discernible differences between the treatment groups.  *“Hindlimb suspended healing rats were subjected to hindlimb unweighting (24 h after surgery), which induced stress reduction by eliminating ground reaction force by using the noninvasive tail suspension protocol of the NASA-Ames center… The health of suspended healing animals was carefully monitored, and*  *no complications with surgery or suspension were observed*.*”* |
| Thornton et al. 2003 | **High risk**  There are visually discernible differences between the treatment groups. |
| Thornton et al. 2005 | **High risk**  There are visually discernible differences between the treatment groups. |
| **Bias in random outcome assessment** | |
| Bray et al. 1992 | **Unclear**  There is no mention of how the animals were ordered during outcome assessment. |
| Burroughs et al. 1990 | **Unclear**  There is no mention of how the animals were ordered during outcome assessment. |
| Gomez et al. 1991 | **Unclear**  There is no mention of how the animals were ordered during outcome assessment. |
| Lechner et al. 1991 | **Unclear**  There is no mention of how the animals were ordered during outcome assessment. |
| Provenzano et al. 2003 | **Unclear**  There is no mention of how the animals were ordered during outcome assessment. |
| Thornton et al. 2003 | **Unclear**  There is no mention of how the animals were ordered during outcome assessment. |
| Thornton et al. 2005 | **High risk**  As an ad-hoc decision to not measure 3-week immobilized isolated MCL ligaments was made following the results of other time intervals, individual specimens were not analysed at random.  *“we elected not to measure the failure*  *load of the isolated MCL* *based on the results collected at 6 and*  *14 weeks.”* |
| **Bias in blinding of outcome assessor** | |
| Bray et al. 1992 | **Low risk**  Automated test where the authors employed a reproducible joint position device and refers to a standardised protocol.  *“A reproducible tibiofemoral test position… established by means of a specially designed joint alignment device… Each MCL complex was subjected to a testing sequence [citation]…”* |
| Burroughs et al. 1990 | **Low risk**  Automated test that refers to previously established measurement methods and is computer-analysed.  *“The grips were set up so that distraction and compression would be applied along the axis of the shaft of the tibia with the femur mounted at 45° to this axis.[citation] Strain gauges were connected through a conditioning amplifier to a storage oscilloscope and via an analog to digital converter to a microcomputer.”* |
| Gomez et al. 1991 | **Low risk**  Automated test that is sufficiently described and is computer-analysed. |
| Lechner et al. 1991 | **Low risk**  Automated test that refers to previously established measurement methods and is computer-analysed.  *“The knee was mounted in a servohydraulic materials-testing machine at 45° of flexion with clamps on the femur and tibia as described by …[citation]. Loading was measured by a strain gauge ring and transmitted to an oscilloscope and, via an analog to digital converter, to a microcomputer.”* |
| Provenzano et al. 2003 | **Low risk**  Automated test that refers to a previously established test protocol.  *“… testing were performed by using methods similar to*  *those previously described [citations].”* |
| Thornton et al. 2003 | **Low risk**  Automated test that refers to a standardised test protocol.  *“Femur-MCL-tibia complexes were dissected, mounted. and tested*  *as per our standard protocol for creep testing (cyclic and static)*  *[citations].”* |
| Thornton et al. 2005 | **Low risk**  Automated test that refers to a standardised test protocol.  *“All groups underwent the same mechanical testing protocol, shown*  *previously to characterize the mechanical behaviour of injured and*  *uninjured control ligaments [citation].”* |
| **Bias due to incomplete outcome data** | |
| Bray et al. 1992 | **Low risk**  Complete outcome data is provided. |
| Burroughs et al. 1990 | **Low risk**  Complete outcome data is provided. |
| Gomez et al. 1991 | **Low risk**  Complete outcome data is provided. |
| Lechner et al. 1991 | **Low risk**  Complete outcome data is provided. |
| Provenzano et al. 2003 | **Low risk**  Complete outcome data is provided. |
| Thornton et al. 2003 | **High risk**  Loss of specimens disproportionally affecting immobilized scars.  *“Six bilateral scars were excluded from this study… two because there was not enough scar tissue to test (one non-immobilized 3-week scar and one nonimmobilized 14-week scar) and four because the creep test stress was*  *programmed incorrectly (one immobilized 3-week scar and three*  *immobilized 6-week scars).”* |
| Thornton et al. 2005 | **High risk**  Loss of specimens disproportionally affecting immobilized scars.  *“…for immobilized group deformation, failure energy and stiffness due to limitations in data collection.”* |
| **Bias due to selective outcome reporting** | |
| Bray et al. 1992 | **Unclear**  No study protocol is available, but the study was conducted prior to the inception of a registry specifically aimed at animal research ([www.preclinicaltrials.eu](http://www.preclinicaltrials.eu)) and prior to the updated ARRIVE guidelines 2.0 (that mentions preregistration as part of their “recommended” set of items). |
| Burroughs et al. 1990 | **Unclear**  No study protocol is available, but the study was conducted prior to the inception of a registry specifically aimed at animal research ([www.preclinicaltrials.eu](http://www.preclinicaltrials.eu)) and prior to the updated ARRIVE guidelines 2.0 (that mentions preregistration as part of their “recommended” set of items). |
| Gomez et al. 1991 | **Unclear**  No study protocol is available, but the study was conducted prior to the inception of a registry specifically aimed at animal research ([www.preclinicaltrials.eu](http://www.preclinicaltrials.eu)) and prior to the updated ARRIVE guidelines 2.0 (that mentions preregistration as part of their “recommended” set of items). |
| Lechner et al. 1991 | **Unclear**  No study protocol is available, but the study was conducted prior to the inception of a registry specifically aimed at animal research ([www.preclinicaltrials.eu](http://www.preclinicaltrials.eu)) and prior to the updated ARRIVE guidelines 2.0 (that mentions preregistration as part of their “recommended” set of items). |
| Provenzano et al. 2003 | **Unclear**  No study protocol is available, but the study was conducted prior to the inception of a registry specifically aimed at animal research ([www.preclinicaltrials.eu](http://www.preclinicaltrials.eu)) and prior to the updated ARRIVE guidelines 2.0 (that mentions preregistration as part of their “recommended” set of items). |
| Thornton et al. 2003 | **Unclear**  No study protocol is available, but the study was conducted prior to the inception of a registry specifically aimed at animal research ([www.preclinicaltrials.eu](http://www.preclinicaltrials.eu)) and prior to the updated ARRIVE guidelines 2.0 (that mentions preregistration as part of their “recommended” set of items). |
| Thornton et al. 2005 | **High risk**  Movement groups were followed up to 40 weeks, but not the immobilized. No clear rationale is given for this.  *“Testing was performed at 3 (n = 12), 6*  *(n = 22), and 14 (n = 24) weeks. The remaining 23 rabbits, which had both limbs non-immobilized (non-immobilized group), were*  *tested at 3 (n = 10), 6 (n = 12), 14 (n = 12), and 40 (n = 12) weeks.”*  Likewise, the authors have reported data on 3-week failure load (Table 3 of their paper) for both immobilization and ad-lib movement, but 3-week information on the other outcomes for immobilization are absent (Table 2). |
| **Bias due to other issues** | |
| Bray et al. 1992 | **High risk**  Deviations from intended interventions.  “The criteria for defining a knee as “immobilized”  were that there was no voluntary joint movement  and less than a few degrees of passive motion: with  these criteria, no animal had a completely rigidly  immobilized knee after surgery. Also, immobilized  animals in later intervals appeared to have greater  passive knee motion on manual examinations compared  to early healing intervals.” |
| Burroughs et al. 1990 | **Low risk**  No apparent flaws stand out. |
| Gomez et al. 1991 | **Low risk**  No apparent flaws stand out. |
| Lechner et al. 1991 | **High risk**  Unit of analysis error – Effect estimates for the study arms were calculated and expressed as ratios to contralateral controls.  *“Laxity of the experimental knee was expressed as the experimental knee joint displacement divided by the control knee displacement… Stiffnes was expressed as a percent by dividing the slope of the MCL load deformation curve in the experimental knee by the slope of the MCL curve in the control knee and multiplying by 100. Likewise, the tensile strength of the experimental MCL was expressed as a percent by dividing the maximum force to failure of the experimental MCL by the control MCL value and multiplying by 100.”* |
| Provenzano et al. 2003 | **Low risk**  No apparent flaws stand out. |
| Thornton et al. 2003 | **High risk**  Deviations from intended interventions.  Cited Bray et al. 1992 regarding immobilization technique employed but made no mention whether the limitations of the technique had been addressed.  *“…right hindlimb was pin-immobilized in full flexion (approximately*  *150-160”) [citation].”* |
| Thornton et al. 2005 | **High risk**  Deviations from intended interventions.  Cited Bray et al. 1992 regarding immobilization technique employed but made no mention whether the limitations of the technique had been addressed.  *“…animals had their right hindlimbs pin-immobilized*  *in full flexion [citation]”* |
